# Clinical Predictors of Mortality in Severe Fever with Thrombocytopenia Syndrome: An Updated Systematic Review and Meta-Analysis

**DOI:** 10.3390/pathogens15070767

**Published:** 2026-07-21

**Authors:** Ke-Xin Wang, Guo-Mei Xia, Yu-Han Liu, Shuai-Ru Jiao, Yu-Feng Gao, Sheng-Qun Deng

**Affiliations:** 1 The Second Affiliated Hospital of Anhui Medical University, Hefei 230601, China; 2313010085@stu.ahmu.edu.cn (K.-X.W.); efy130401@fy.ahmu.edu.cn (G.-M.X.); 2 Department of Pathogen Biology, School of Basic Medical Sciences, Anhui Medical University, Hefei 230032, China; 2135010026@stu.ahmu.edu.cn (Y.-H.L.); 2545010058@stu.ahmu.edu.cn (S.-R.J.); 3 Anhui Province Key Laboratory of Zoonoses, The Provincial Key Laboratory of Zoonoses of High Institutions in Anhui, Hefei 230032, China; 4 Department of Infectious Diseases, The First Affiliated Hospital of Anhui Medical University, Hefei 230022, China; 5 Anhui Province Key Laboratory of Infectious Diseases, Anhui Medical University, Hefei 230032, China

**Keywords:** severe fever with thrombocytopenia syndrome, SFTS, *Bandavirus dabieense*, mortality, risk factors, meta-analysis, neurological manifestations, complications

## Abstract

Severe fever with thrombocytopenia syndrome (SFTS) is an emerging tick-borne disease with a high case fatality rate and no specific treatment. Identifying robust clinical predictors of mortality is crucial for improving patient outcomes. This systematic review and meta-analysis aimed to comprehensively evaluate the association of underlying diseases, hemorrhagic symptoms, neurological signs, and complications with fatal outcomes in SFTS patients. We systematically searched PubMed, Web of Science, CNKI, and Wan Fang databases up to 1 March 2026 for observational studies. Pooled odds ratios (ORs) with 95% confidence intervals (CIs) were calculated using fixed- or random-effects models. Forty-five studies, encompassing 8078 patients (1729 deaths), were included. Underlying diabetes (OR = 1.77) and hypertension (OR = 2.38) were significant risk factors. Among hemorrhagic symptoms, systemic manifestations (OR = 4.19), melena (OR = 4.26), and petechiae (OR = 3.20) were strong predictors. Neurological symptoms, particularly coma (OR = 55.07), were the most powerful predictors of death. Complications like multiple organ dysfunction syndrome (MODS, OR = 19.77) and disseminated intravascular coagulation (DIC, OR = 12.02) also significantly increased mortality risk. The findings confirm that a range of clinical features, from underlying conditions to severe neurological and organ complications, are strongly associated with SFTS mortality. These predictors can guide clinicians in early risk stratification and intensive monitoring, especially in high-risk patients, to potentially reduce the high case-fatality rate.

## 1. Introduction

Severe fever with thrombocytopenia syndrome (SFTS) is a newly recognized tick-borne infectious disease caused by *Bandavirus dabieense*, a pathogen previously designated SFTS virus (SFTSV) [1]. The first discovery of SFTSV occurred in 2009 in rural central and eastern China [2]; since then, it has been reported in multiple countries, notably the Republic of Korea [3], Japan [4], Myanmar [5], and Thailand [6]. In Kenya and Pakistan, SFTS-specific antibodies have been detected, suggesting possible exposure to the virus [7,8]. This virus is classified as a member of the genus *Bandavirus*, within the family *Phenuiviridae*, order *Hareavirales* [9]. The tick species *Hemaphysalis longicornis* is the principal reservoir and vector of *Bandavirus dabieense*, with human infection occurring mainly via tick bites [10]. In recent years, additional potential transmission routes have been documented, such as human-to-human [11], animal-to-human [12], and even aerosol transmission [13]. The expansion of known transmission pathways raises concerns about the potential for wider, including pandemic, spread of SFTSV globally [14]. Despite this growing threat, no effective vaccines or specific therapeutics are currently available [15]. Although SFTS has been removed from the latest World Health Organization (WHO) priority pathogen list, it remains a disease that cannot be ignored and still requires active prevention and control efforts.

Patients with SFTS commonly present with acute high fever, profound fatigue and gastrointestinal symptoms [16]. Neurological signs such as confusion, seizures, or coma indicate severe disease [17]. Characteristic laboratory findings include marked thrombocytopenia and leukopenia [18]. In accordance with the principles of critical care medicine, early intervention in a patient’s clinical trajectory can help prevent progression to critical stages. Therefore, identifying and summarizing clinical indicators that may signal a poor prognosis is essential, as it enables more focused care and may help reduce mortality.

SFTS has been explored in multiple studies. Previous meta-analyses have investigated clinical symptoms and laboratory parameters; however, several limitations remain. First, prior meta-analyses have largely centered on non-specific clinical features and laboratory abnormalities, without providing a comprehensive synthesis of clinically critical severe complications and diverse neurological presentations [19], which consequently hampers the clinical applicability of their results. Additionally, some indicators, such as systemic hemorrhagic manifestations and acute respiratory distress syndrome (ARDS), have not been analyzed, which may lead to the neglect of key clinical warning signs in practice. Second, although neurological involvement has consistently been identified as a predictor of poor prognosis, previous reviews generally analyzed neurological manifestations as broad categories without separately evaluating clinically important manifestations such as coma, seizures, tremor, convulsion and mental status changes [20,21]. Third, previous studies primarily summarized whether individual predictors were associated with mortality, but did not establish the relative hierarchy of clinical predictors according to their prognostic strength, limiting their utility for bedside risk stratification [22]. Finally, multiple large observational studies have been published during the past three years, making an updated evidence synthesis necessary.

Therefore, our research aims to systematically assess the relationships of chronic underlying diseases, bleeding symptoms, severe complications and specific neurological symptoms with the risk of fatal outcomes in SFTS patients. By incorporating recently published studies, we intend to provide evidence for constructing a more precise and clinically actionable risk stratification model.

## 2. Materials and Methods

### 2.1. Protocol and Registration

The protocol was registered with PROSPERO in April 2026 (CRD420261362310).

### 2.2. Search Strategy and Selection Criteria

We conducted a systematic review and meta-analysis in accordance with PRISMA guidelines [23]. Literature databases, including PubMed, Web of Science, CNKI, and the Chinese Wan Fang Database, were searched. In the search, we used the following search terms and keywords in Chinese and English: (“Severe fever with thrombocytopenia syndrome” or “SFTS” or “SFTSV” or “*Bandavirus dabieense*”) and (“death” or “survival” or “risk factors” or “poor outcomes” or “fatal” or “nonfatal”). We proactively searched gray literature, and for studies lacking necessary data in publications, we contacted the authors directly and obtained the original datasets for analysis. The search was updated until 1 March 2026. The PRISMA Checklist for this systematic review is provided in Supplementary S1.

If a study met all the following criteria, it was included in the meta-analysis:

1. Enrolled patients fulfilled at least one of the following conditions: (I) isolation of the virus from patient samples; (II) a minimum fourfold increase in specific antibody titers between paired acute-phase and convalescent-phase serum samples; or (III) detection of SFTSV RNA in serum through reverse-transcriptase polymerase chain reaction (RT-PCR).
2. The clinical outcomes were classified as “nonfatal” or “fatal”;
3. Research reported clinical symptoms and mortality risk and provided the initial number of cases;
4. The research design was observational (cohort or case–control studies);

Studies were excluded if they met any of the following conditions: duplicate publications, systematic reviews and meta-analyses, animal experiments, case reports, reply letters, non-English or Chinese literature, conference abstracts, and studies with unsuitable designs, objectives, or insufficient relevant data.

### 2.3. Data Abstraction

Two investigators independently screened the studies, extracted the data, and cross-verified the findings. Any conflicting conclusions were settled by consensus or the involvement of a third reviewer. We discarded the studies that failed to meet the inclusion criteria and extracted the following information from the remaining studies: (1) publication details (e.g., article title, first author, publication year, data collection period, region, and study type); (2) case numbers (survivors and non-survivors); and (3) original 2 × 2 table data of relevant clinical symptoms.

### 2.4. Quality Assessment

We used the Newcastle–Ottawa Quality Assessment Scale (NOS) to evaluate the quality of the included studies. Studies are rated as high quality for scores ranging from 7 to 9 points, moderate quality for scores ranging from 4 to 6 points, and low quality for scores ranging from 0 to 3 points. Two researchers completed the evaluation and settled any disagreements by consulting a third investigator.

### 2.5. Statistical Analysis

We used Review Manager 5.4 and STATA version 16 to perform the meta-analysis. The associations between clinical manifestations and patient outcomes were estimated by calculating ORs along with their 95% CIs, and the results were visually presented via forest plots. Heterogeneity among studies was quantified using the I^2^ statistic. A random-effects model was applied when I^2^ was ≥50%, indicating statistically significant heterogeneity [24]; otherwise, a fixed-effects model was selected [25].

To explore potential reasons for heterogeneity, subgroup analyses were performed by study site (single- or multi-center) and country (China, South Korea and Japan). Furthermore, we conducted a sensitivity analysis to assess the stability of the pooled results. For outcomes involving five or more studies, we assessed publication bias by using funnel plots and performing Egger’s test, with a *p* value of less than 0.05 considered to indicate significant bias. The trim-and-fill method was used to correct the bias.

## 3. Results

### 3.1. Search Results

**Figure 1:**
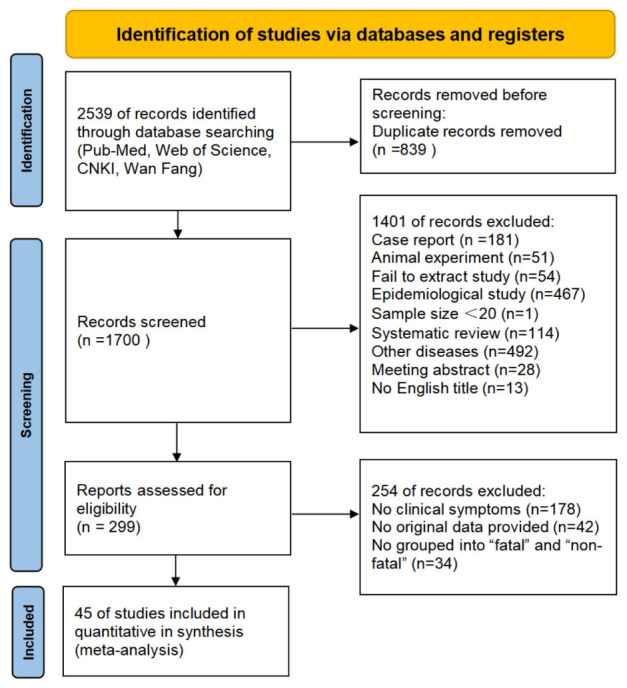
Flow chart of the study selection process in this meta-analysis.

We identified 2539 studies through preliminary retrieval. Afterward, we excluded 839 studies to remove duplicates. During the preliminary screening phase, we excluded 853 studies that did not meet the inclusion criteria on the basis of title and abstract, including systematic reviews, animal experiments, guidelines, non-Chinese/English publications, and case reports. Furthermore, we excluded 548 studies because of irrelevance of the topic or inappropriate study design. We screened the remaining full texts and excluded another 254 studies because of a lack of key clinical data. Finally, we included 45 studies in our study (Figure 1).

The basic characteristics and Newcastle–Ottawa scores (NOSs) of the studies included in this study are displayed in the chart [17,23,24,25,26,27,28,29,30,31,32,33,34,35,36,37,38,39,40,41,42,43,44,45,46,47,48,49,50,51,52,53,54,55,56,57,58,59,60,61,62,63,64,65,66,67,68,69]. We included 45 articles in our study, comprising 8078 SFTS patients, of whom 6349 survived and 1729 died. All of the studies were conducted in the Western Pacific region, with 91.1% (41/45) from China, 6.7% (3/45) from South Korea, and 2.2% (1/45) from Japan. On the basis of the NOS assessment, all included studies scored above 7, indicating high quality and thus meeting the fundamental criteria for meta-analysis (Table 1).

### 3.2. Association of Underlying Diseases with SFTS Mortality

**Figure 2:**
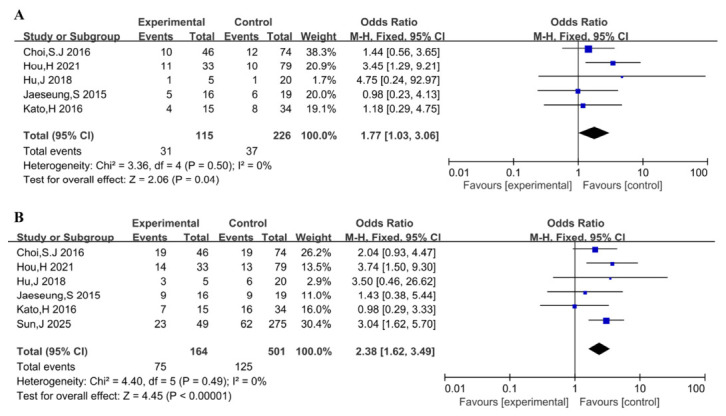
Forest plot of underlying diseases associated with fatal SFTS: diabetes (**A**) and hypertension (**B**).

Five studies on diabetes (I^2^ = 0%, *p* = 0.50) and six studies on hypertension (I^2^ = 0%, *p* = 0.49) were included. The fixed effects model revealed that diabetes (OR = 1.77; 95% CI: 1.03–3.06; *p* = 0.04) and hypertension (OR = 2.38; 95% CI: 1.62–3.49; *p* < 0.001) were related to high mortality among SFTS patients (Figure 2).

### 3.3. Association of Hemorrhagic Symptoms with SFTS Mortality

**Figure 3:**
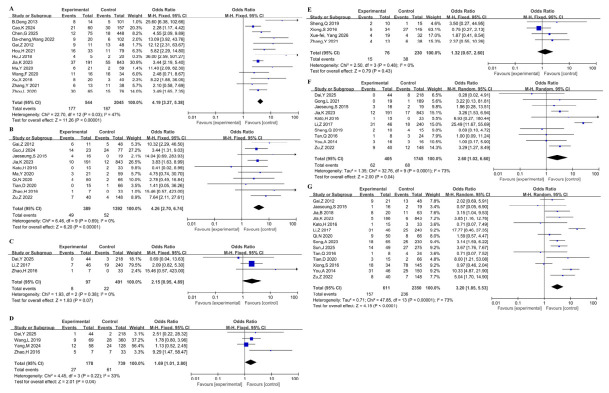
Forest plot of hemorrhagic symptoms associated with fatal SFTS: systemic hemorrhagic manifestations (**A**), melena (**B**), hematemesis (**C**), subcutaneous hemorrhage (**D**), gingival bleeding (**E**), ecchymoses (**F**) and petechiae (**G**).

Thirteen studies on systemic hemorrhagic manifestations (I^2^ = 47%, *p* = 0.03), twelve studies on melena (I^2^ = 0%, *p* = 0.69), three studies on hematemesis (I^2^ = 0%, *p* = 0.38), four studies on subcutaneous hemorrhage (I^2^ = 33%, *p* = 0.22), ten studies on gingival bleeding (I^2^ = 73%, *p* < 0.001), four studies on ecchymoses (I^2^ = 0%, *p* = 0.48), and fourteen studies on petechiae (I^2^ = 73%, *p* < 0.001) were included. According to the fixed-effects model, systemic hemorrhagic manifestations (OR = 4.19; 95% CI: 3.27–5.38; *p* < 0.001), melena (OR = 4.26; 95% CI: 2.70–6.74; *p* < 0.001), petechiae (OR = 3.20; 95% CI: 1.85–5.53; *p* < 0.001), and subcutaneous hemorrhage (OR = 1.69; 95% CI: 1.01–2.80; *p* = 0.04) significantly increased the odds of mortality among SFTS patients. Similarly, gingival bleeding was correlated with increased mortality in the random-effects analysis (OR = 2.60, 95% CI: 1.02–6.60; *p* = 0.04). In contrast, ecchymoses (OR = 1.32; 95% CI: 0.67–2.60; *p* = 0.43) and hematemesis (OR = 2.15; 95% CI: 0.95–4.89; *p* = 0.07) were not significantly different (Figure 3).

### 3.4. Association of Neurological Symptoms with SFTS Mortality

**Figure 4:**
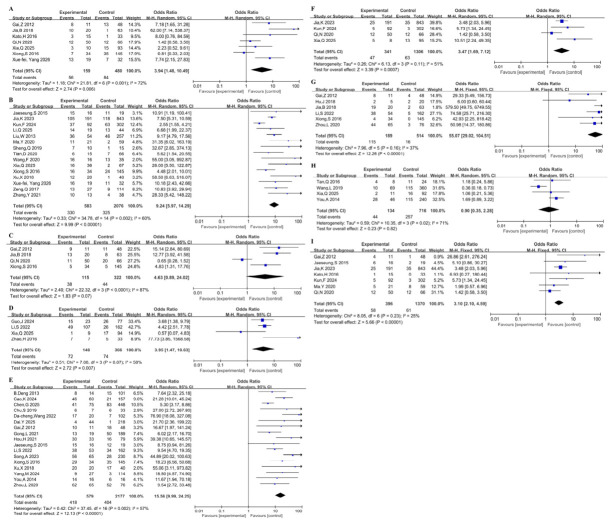
Forest plot of neurological symptoms associated with fatal SFTS, including tremor (**A**), disturbance of consciousness (**B**), lethargy (**C**), mental state changes (**D**), systemic neurological manifestations (**E**), seizures (**F**), coma (**G**), headache (**H**) and convulsions (**I**).

Seven studies on tremor (I^2^ = 72%, *p* = 0.001), fifteen studies on disturbance of consciousness (I^2^ = 60%, *p* = 0.002), four studies on lethargy (I^2^ = 87%, *p* < 0.001), four studies on mental state change (I^2^ = 58%, *p* = 0.07), seventeen studies on systemic neurological manifestations (I^2^ = 57%, *p* = 0.002), four studies on seizures (I^2^ = 51%, *p* = 0.11), six studies on coma (I^2^ = 37%, *p* = 0.16), four studies on headache (I^2^ = 71%, *p* = 0.02) and seven studies on convulsion (I^2^ = 25%, *p* = 0.23) were included. Coma (OR = 55.07; 95% CI: 29.02–104.51; *p* < 0.001) and convulsions (OR =3.10; 95% CI: 2.10–4.59; *p* < 0.001) emerged as significant predictors of mortality among SFTS patients according to the fixed-effects model. The presence of tremors (OR = 3.94; 95% CI: 1.48–10.49; *p* = 0.006), disturbances in consciousness (OR = 9.24; 95% CI: 5.97–14.29; *p* < 0.001), mental state changes (OR = 3.95; 95% CI: 1.47–10.63; *p* = 0.007), systemic neurological manifestations (OR = 15.56; 95% CI: 9.99–24.25; *p* < 0.001), and seizures (OR = 3.47; 95% CI: 1.69–7.12; *p* < 0.001) were strongly linked to increased mortality risk in patients according to the random effects model (Figure 4). However, lethargy (OR = 4.63; 95% CI: 0.89–24.02; *p* = 0.07) and headache (OR = 0.90; 95% CI: 0.35–2.28; *p* = 0.82) were not significantly different.

### 3.5. Association of Complications with SFTS Mortality

**Figure 5:**
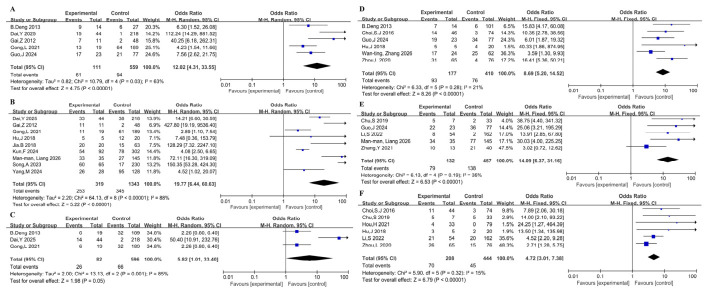
Forest plot of complications associated with fatal SFTS: disseminated intravascular coagulation (DIC) (**A**), multiple organ dysfunction syndrome (MODS) (**B**), acute respiratory distress syndrome (ARDS) (**C**), renal injury (**D**), heart failure (**E**) and arrhythmia (**F**).

Five studies on disseminated intravascular coagulation (DIC) (I^2^ = 63%, *p* = 0.03), nine studies on multiple organ dysfunction syndrome (MODS) (I^2^ = 88%, *p* < 0.001), three studies on acute respiratory distress syndrome (ARDS) (I^2^ = 85%, *p* = 0.001), five studies on heart failure (I^2^ = 35%, *p* = 0.19), six studies on renal injury (I^2^ = 21%, *p* = 0.28), and six studies on arrhythmia (I^2^ = 15%, *p* = 0.32) were included. According to the random effects analysis, DIC (OR = 12.02; 95% CI: 4.31–33.55; *p* < 0.001), MODS (OR = 19.77; 95% CI: 6.44–60.63; *p* < 0.001) and ARDS (OR = 5.82; 95% CI: 1.01–33.40; *p* = 0.05) emerged as significant risk factors for death. In the fixed-effects model, heart failure (OR = 14.09; 95% CI: 6.37–31.16; *p* < 0.001), renal injury (OR = 8.69; 95% CI: 5.20–14.52; *p* < 0.001) and arrhythmia (OR = 4.72; 95% CI: 3.01–7.38; *p* < 0.001) were associated with increased mortality among SFTS patients (Figure 5).

### 3.6. Subgroup Analyses

**Figure 6:**
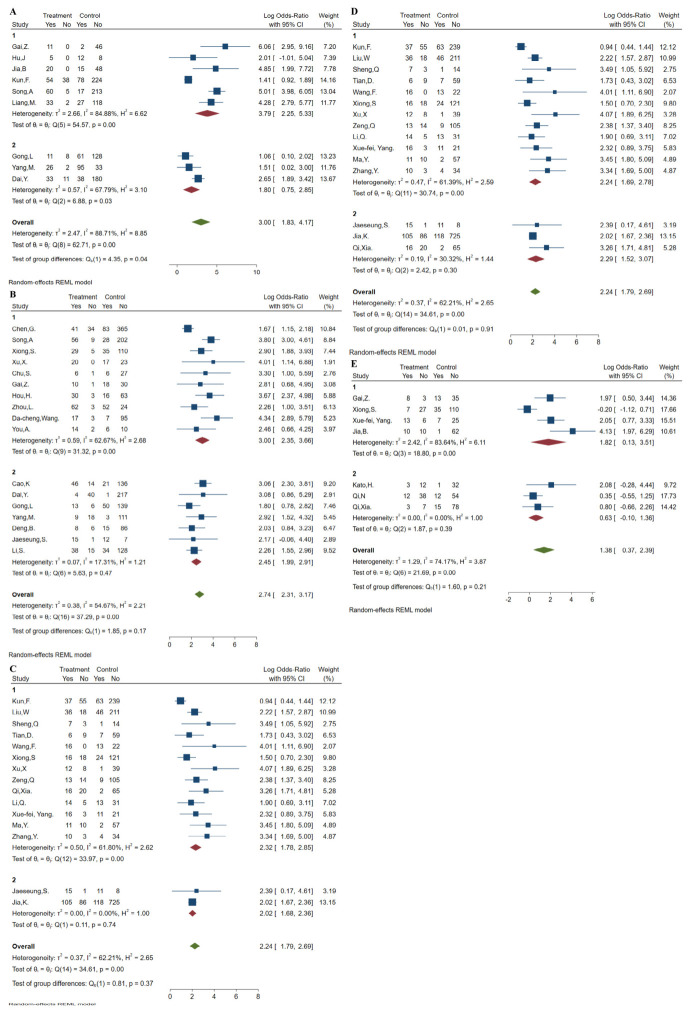
Subgroup analyses for SFTS mortality predictors: (**A**) MODS by type; (**B**) systemic neurological manifestations by type; (**C**) disturbance of consciousness by country; (**D**) disturbance of consciousness by type; (**E**) tremor by type.

To explore potential reasons for heterogeneity, subgroup analyses were performed by country and study site. In single-center studies, MODS (OR = 19.77, 95% CI: 2.25–5.33), systemic neurological manifestations (OR = 3.00, 95% CI: 2.35–3.66), and disturbance of consciousness (OR = 2.24, 95% CI: 1.69–2.78) were considered effective predictors of increased mortality in patients with SFTS. In multi-center studies, systemic neurological manifestations (OR = 2.74, 95% CI: 2.31–3.17) and disturbance of consciousness (OR = 2.29, 95% CI: 1.52–3.07) increased SFTS mortality. Moreover, disturbance of consciousness was a good predictor of poor SFTS prognosis in China (OR = 2.32, 95% CI: 1.78–2.85) as well as in South Korea (OR = 2.02, 95% CI: 1.68–2.36) (Figure 6). However, for these indicators with significant heterogeneity, subgroup analyses did not reduce the heterogeneity to less than 50% in either subgroup simultaneously, indicating that country and study site were not the sources of heterogeneity. For other indicators with high heterogeneity, subgroup analyses could not be performed because of the limited number of studies reporting them or the consistent characteristics of the included studies.

### 3.7. Sensitivity Analyses and Publication Bias

**Figure 7:**
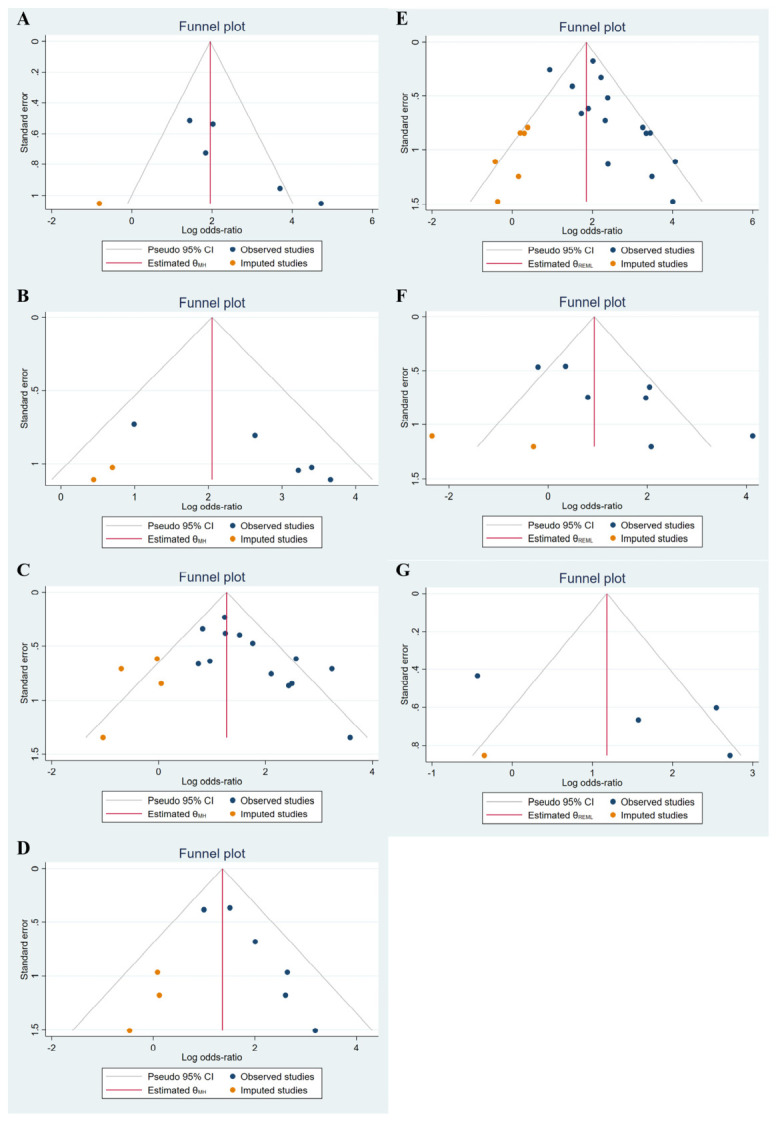
Funnel plots with trim-and-fill adjustment for publication bias (imputed studies added to the left): (**A**) DIC; (**B**) heart failure; (**C**) systemic hemorrhagic manifestations; (**D**) arrhythmia; (**E**) disturbance of consciousness; (**F**) tremor; (**G**) lethargy.

We conducted leave-one-out sensitivity analyses, and all of the included studies consistently supported the pooled effect size, confirming the stability of the findings (Figures S1–S4). Publication bias was evaluated using Egger’s regression test, which revealed significant bias in disturbance of consciousness (*p* = 0.017), tremor (*p* < 0.001), hemorrhagic manifestations (*p* < 0.001), lethargy (*p* < 0.05), DIC (*p* < 0.05), heart failure (*p* = 0.021) and arrhythmia (*p* = 0.0325). After correction, the ORs and 95% CIs for all the predictors except arrhythmia and tremor remained unchanged (Figure 7).

## 4. Discussion

Our study provided an updated synthesis of evidence from 45 studies involving 8078 patients. The mortality risk associated with specific underlying diseases (diabetes, hypertension), hemorrhagic symptoms (systemic hemorrhagic manifestations, melena, petechiae, subcutaneous hemorrhage and gingival bleeding), neurological symptoms (systemic neurological manifestations, disturbance of consciousness, convulsions, tremors, mental state changes, seizures and coma), and severe complications (DIC, MODS, ARDS, arrhythmia, heart failure, and renal injury) was assessed. By incorporating recent studies, our study offered a contemporary and comprehensive overview of clinical predictors of SFTS mortality.

Our analysis extends prior systematic reviews that identified underlying diseases as general risk factors for SFTS mortality [20] by further specifying individual conditions. We found that diabetes mellitus and hypertension independently predicted fatal outcomes, a result that aligns with those of previous reports [70]. However, these associations were not observed in the study by Wang et al., underscoring the remaining inconsistencies across meta-analyses [22]. It has been reported that hyperglycemia can impair immune responses and significantly alter the equilibrium of pro- and anti-inflammatory cytokines, thereby restricting immune cell function [71]. Additionally, elevated blood glucose leads to osmotic diuresis, hypovolemia, and electrolyte loss [72]. Furthermore, hyperglycemia can trigger the accumulation of advanced glycation end products, whose interaction with cell membranes may lead to endothelial dysfunction. This mechanism is supported by animal studies showing a significant correlation between endothelial cell damage and elevated mortality risk [73]. Moreover, research suggests that hypertensive patients have lower cardiac reserve, which may explain why hypertension is a high-risk factor [47].

As SFTS is a relatively common symptom in SFTS patients, a consensus has been reached on its ability to predict systemic hemorrhagic manifestations [16,17]. Our study also revealed the same result. In line with previous studies [40,43], we identified melena as a predictor of mortality, a relationship that underscores the role of upper gastrointestinal bleeding in disease progression. Moreover, the observed link between subcutaneous hemorrhage and fatal outcomes aligns with that reported by Wang et al. [20], further validating it as a reliable prognostic marker. We reaffirmed the link between gingival bleeding and mortality, a result that mirrors the results reported in previous studies [43,46]. However, Gong et al. [26] reported no significant association for gingival bleeding, possibly owing to the greater statistical power achieved through meta-analysis. Hematemesis showed no significant predictive value for mortality in our analysis, which aligns with the findings of Choi et al. [30] but diverges from those of Deng et al. [39] and Zhao et al. [57], underscoring the need for additional studies to clarify this discrepancy. However, ecchymosis was not significantly associated in our study, which is consistent with the findings of Wang et al. [22]. The underlying mechanism may involve a virus-induced systemic inflammatory response (SIRS), direct injury to vascular endothelial cells leading to enhanced permeability, thrombocytopenia, or extensive consumption of coagulation factors [74,75].

Among all the clinical manifestations, neurological symptoms demonstrated the strongest predictive power for mortality.

In patients aged 50 years or younger, only neurological manifestations and dyspnea were linked to a fatal outcome [16]. Moreover, the risk of death increased with increasing number of neurological symptoms, and a clear dose–response trend was observed in the corresponding odds ratios [16]. Our study revealed that the presence of systemic neurological abnormalities, as well as specific symptoms such as tremors, disturbances of consciousness, mental state changes, seizures, coma and convulsion, significantly contributes to death in SFTS patients, which is consistent with the findings of independent studies [16,53,61]. However, in our study, lethargy did not significantly predict poor prognosis, which differs from the findings of Wang et al. [22] but is consistent with Deng et al. [39]. This discrepancy may be attributed to limitations in sample size as well as substantial heterogeneity. Our analysis revealed that nonspecific neurological manifestations such as headache could not serve as effective predictors, which is consistent with the findings of the study by Li [16]. SFTSV nucleic acid has been detected in the cerebrospinal fluid (CSF) of encephalitis or encephalopathy patients, with a positivity rate reaching 75%, suggesting that direct viral invasion of the central nervous system may be a key pathological mechanism. Furthermore, in some cases, the viral load in the CSF is comparable to the serum level, suggesting that the virus can cross the blood–brain barrier or enter the CNS through infected leukocytes [76]. In summary, neurological damage often indicates critical disease progression and poor prognosis, underscoring the importance of close neurological monitoring throughout the disease course.

Finally, the occurrence of complications, particularly multiorgan involvement, represents the progression of the disease to its most critical stage. Existing evidence has indicated this perspective [26,32,40,41]. In our study, complications such as ARDS, DIC, MODS, renal injury, heart failure and arrhythmia emerged as strong predictors of SFTS mortality, with high ORs. Numerous studies have shown that SFTSV is pantropic and can directly infect and damage parenchymal cells of various tissues and organs, including hepatocytes, cardiomyocytes, renal tubular epithelial cells, and neurons [26]. This direct injury constitutes the initiating step in the development of MODS. Animal model studies have confirmed that the kidneys and liver are the primary target organs of SFTSV [77]. Furthermore, viral replication and cellular damage release many damage-associated molecular patterns (DAMPs), stimulating the innate immune system and triggering an excessive inflammatory response known as a cytokine storm. Deng et al. reported that this systemic inflammatory state not only aggravates primary organ injury but also leads to dysfunction of distal organs through circulating inflammatory mediators [39]. Organ dysfunctions do not exist in isolation; rather, they exacerbate each other through hemodynamic, metabolic, and inflammatory mediators and ultimately form MODS. Both Gai et al. [40] and He et al. [78] reported that 7–13 days of the disease course represents a critical window for disease progression. Jie et al. reported that the presence or persistence of MODS within 72 h of admission is closely associated with an inpatient mortality rate as high as 76% [79]. These findings suggest that if severe early interplay among organs is not effectively intercepted during this critical period, multiple organ failure and death can occur irreversibly. Therefore, timely detection and targeting of key links in this chain of reactions are crucial for improving the prognosis of SFTS.

Compared with previous meta-analyses, the present study expands the current evidence in several important ways. First, by incorporating 45 studies with 8078 patients, we substantially updated the evidence base through recently published large observational cohorts. Second, moving beyond non-specific clinical features and routine laboratory abnormalities, we systematically synthesized severe complications, hemorrhagic manifestations, underlying diseases, and detailed neurological symptoms, thereby strengthening the clinical relevance of the findings. Third, unlike previous meta-analyses that mainly evaluated broad categories of clinical manifestations, the present study further analyzed individual neurological and hemorrhagic manifestations separately. This more granular approach allowed the prognostic value of specific clinical features, such as coma, disturbance of consciousness, ecchymosis, and petechiae, to be evaluated individually, thereby providing evidence that is more readily applicable in clinical practice. Fourth, this study also systematically evaluated several clinically important manifestations that have not been quantitatively synthesized in previous meta-analyses, such as acute respiratory distress syndrome (ARDS) and systemic hemorrhagic manifestations. By confirming their associations with poor prognosis in SFTS, our findings further complement the existing evidence and provide a more comprehensive basis for mortality risk assessment.

Several limitations of this study warrant consideration when interpreting the results. First, significant heterogeneity was observed in several analyses. Although we explored potential sources through sensitivity and subgroup analyses, the possibility of unmeasured confounding factors cannot be entirely ruled out; furthermore, some subgroup analyses included only one study because of the limited number of studies, restricting the explanation of heterogeneity. Second, as all the data were derived from Asian cohorts, caution is needed when these results are applied to other regions or ethnic groups. Finally, age, as an important factor that may influence disease prognosis, was not subjected to further processing and analysis, which may have affected our interpretation of the results to some extent.

## 5. Conclusions

Our study confirmed that underlying conditions, clinical manifestations, neurological symptoms, and complications are important predictors of death among SFTS patients. In clinical practice, we need to provide closer monitoring for patients who exhibit such symptoms. In high-risk areas, health education and follow-up are necessary to reduce case fatality. Future studies should be conducted in diverse populations, and further elucidation of the immunological and pathophysiological pathways through which comorbidities influence SFTS prognosis is needed.

## Figures and Tables

**Table 1:** Characteristics of the studies included in the meta-analysis.

Study	Type of Study (Prospective/Retrospective) (Case–Control/Cohort Study)	Country	Sites of Study	Study Period	Age (Fatal)	Age (Non-Fatal)	Number (Male/Female)	Fatal	Survival	NOS
Chen, G. (2025)	Retrospective cohort study	China	single-center	2020.01–2023.12	71.0 (66.0–76.0) a	67.0 (57.0–72.0) a	523 (241/282)	75	448	8
Gong, L. (2021)	Retrospective cohort study	China	multi-center	2015.01–2018.06	70.0 (62.0–75.0) a	64.0 (53.0–71.5) a	208 (104/104)	19	189	8
Cao, K. (2024)	Retrospective cohort study	China	multi-center	2021.03–2023.11	69.0 (63.3–75.0) a	63.0 (53.0–69.0) a	217 (92/125)	60	157	7
Xia, Q. (2025)	Retrospective cohort study	China	single-center	2021.04–2024.11	\	\	103	18	85	7
Yang, M. (2024)	Retrospective cohort study	China	multi-center	2016.04–2022.04	70.4 ± 10.8 b	62.8 ± 12.6 b	156 (67/89)	28	128	8
Choi, S.J. (2016)	Retrospective cohort study	South-Korea	multi-center	2013.01–2015.12	73.5 (66.0–79.0) a	66.0 (52.0–74.0) a	120 (61/59)	46	74	7
Song, A. (2023)	Retrospective cohort study	China	single-center	2017.01–2021.12	\	\	295 (145/150)	65	230	7
Dai, Y. (2025)	Retrospective cohort study	China	multi-center	2023.01–2024.07	\	\	262 (104/158)	44	218	7
Li, S. (2025)	Retrospective cohort study	China	single-center	2023.04–2024.08	69.0 (64.0–74.0) a	62.0 (55.0–70.0) a	269 (132/137)	107	162	7
Yuan-yuan Hu (2026)	Retrospective cohort study	China	single-center	2019.01–2025.06	\	\	629	119	510	7
Wan-ting, Zhang. (2026)	Retrospective cohort study	China	single-center	2020.06–2024.06	\	\	86 (50/36)	24	62	7
Li, Q. (2025)	Retrospective cohort study	China	multi-center	2014–2024	78.63 ± 10.01 b	64.58 ± 26.39 b	63 (55/18)	19	44	8
Man-man, Liang. (2026)	Retrospective cohort study	China	single-center	2020.01–2024.11	\	\	180 (75/105)	35	145	7
Xue-fei, Yang. (2026)	Retrospective cohort study	China	single-center	2024.05–2024.07	\	\	51 (30/21)	19	32	7
Deng, B. (2013)	Retrospective cohort study	China	multi-center	2010.06–2011.12	\	\	115 (75/40)	14	101	8
Gai, Z. (2012)	Retrospective cohort study	China	single-center	2008.05–2011.07	64.0 c	61.0 c	59 (29/30)	11	48	8
Li, S. (2022)	Retrospective cohort study	China	multi-center	2015.01–2019.12	71.06 ± 9.58 b	65.04 ± 10.58 b	216 (117/99)	54	162	7
Kun, F. (2024)	Retrospective cohort study	China	single-center	2012–2021	70.45 ± 7.76 b	64.22 ± 10.34 b	394 (189/205)	92	302	7
Jia, K. (2023)	Retrospective cohort study	South-Korea	multi-center	2018–2022	\	\	1034	191	843	8
Da-cheng, Wang. (2022)	Retrospective cohort study	China	single-center	2017–2020	68.1 ± 10.7 b	60.4 ± 13.1 b	122 (64/58)	20	102	8
Xu, X. (2018)	Retrospective cohort study	China	single-center	2014.01–2015.12	68.21 ± 10.40 b	63.95 ± 9.41 b	60	20	40	8
Zu, Z. (2022)	Retrospective cohort study	China	multi-center	2011–2020	72.0 (52.0–88.0) d	64.0 (33.0–88.0) d	188 (102/86)	40	148	7
Sun, J. (2025)	Retrospective cohort study	China	single-center	2021.01–2023.12	70.0 (66.0–76.0) a	65 (56.0–71.0) a	324 (152/172)	49	275	8
Hou, H. (2021)	Retrospective cohort study	China	single-center	2018–2020	64.77 e	56.71 e	112 (58/54)	33	79	7
Guo, J. (2024)	Retrospective cohort study	China	single-center	2022.03–2023.08	65.58 ± 12.21 b	63.11 ± 10.54 b	100 (40/60)	23	77	7
Liu, W. (2013)	Retrospective cohort study	China	single-center	2011–2012	66.0 (34.0–85.0) d	60.0 (7.0–87.0) d	311 (140/171)	54	257	7
Hu, J. (2018)	Retrospective cohort study	China	single-center	2014.01–2017.04	64.40 ± 2.70 b	56.15 ± 13.66 b	25 (15/10)	5	20	7
Sheng, Q. (2019)	Retrospective cohort study	China	multi-center	2011.08–2017.12	66.0 (63.0–72.0) a	60.0 (57.0–67.0) a	25 (15/10)	10	15	7
Jaeseung, S. (2015)	Retrospective cohort study	South-Korea	multi-center	2013	73.5 (62.0–82.0) d	61 (28.0–84.0) d	35 (17/18)	16	19	8
Wang, F. (2020)	Retrospective cohort study	China	single-center	2013.01–2019.01	67.5 (50.0–80.0) d	53.0 (30.0–79.0) d	51 (27/24)	16	35	7
Xiong, S. (2016)	Retrospective cohort study	China	single-center	2015.03–2015.11	63.0 (48.0–78.0) d	57.0 (27.0–91.0) d	179 (71/108)	34	145	8
Tian, D. (2020)	Retrospective cohort study	China	multi-center	2015–2018	67.0 (63.0–77.0) a	65 (55.0–72.0) a	81 (40/41)	15	66	7
Zhao, H. (2016)	Retrospective cohort study	China	single-center	2010.10–2014.08	\	\	40 (19/21)	7	33	7
Zhou, L. (2020)	Retrospective cohort study	China	single-center	2013.05–2019.10	71.0 (62.0–79.0) a	64.0 (56.0–70.0) a	141 (73/68)	65	76	7
Zeng, Q. (2017)	Retrospective cohort study	China	single-center	2011.06–2016.06	68.5 ± 11.6 b	65.0 ± 10.4 b	107 (41/66)	17	90	8
Chu, S. (2019)	Retrospective cohort study	China	single-center	2012.05–2018.05	\	\	40 (19/21)	7	33	7
Jia, B. (2018)	Retrospective cohort study	China	single-center	2010.10–2016.10	68.0 (59.0–71.0) a	58.0 (49.0–63.0) a	83	20	63	8
Kato, H. (2016)	Retrospective cohort study	Japan	multi-center	2013.03–2014.09	85 (79–87) a	69 (62–78) a	48 (17/31)	15	33	7
Qi, N. (2020)	Retrospective cohort study	China	multi-center	2014–2019	66 ± 9.1 b	62 ± 8.8 b	116 (59/57)	50	66	8
Ma, Y. (2020)	Retrospective cohort study	China	single-center	2019.01–2020.06	63.14 ± 8.50 b	60.71 ± 9.77 b	80 (33/47)	21	59	7
Li, Z. (2017)	Retrospective cohort study	China	multi-center	2010.01–2015.12	\	\	286 (142/144)	46	240	8
You, A. (2014)	Retrospective case–control study	China	single-center	2012	63.50 ± 11.37 b	63.44 ± 12.04 b	32	16	16	7
Tan, Q. (2016)	Retrospective case–control study	China	single-center	2013–2014	66.00 ± 12.44 b	66.33 ± 16.64 b	32 (12/20)	8	24	7
Zhang, Y. (2021)	Retrospective cohort study	China	single-center	2020.04–2020.07	\	\	51	13	38	7
Wang, L. (2019)	Prospective cohort study	China	multi-center	2011.04–2018.12	68.3 ± 11.0 b	59.3 ± 11.7 b	429	69	360	8

^a^ Age: median (interquartile range, IQR); ^b^ Age: mean ± standard deviation (SD); ^c^ Age: mean only (no SD reported); ^d^ Age: median (range); NOS: Newcastle–Ottawa Scale (range 0–9; scores ≥ 7 indicate high quality).

## Data Availability

No new data were created or analyzed in this study. Data sharing is not applicable.

## Supplementary Materials

The following supporting information can be downloaded at https://www.mdpi.com/article/10.3390/pathogens15070767/s1; Figure S1: Sensitivity analysis (leave-one-out) for underlying diseases: diabetes (A); hypertension (B). Figure S2: Sensitivity analysis (leave-one-out) for hemorrhagic symptoms: hematemesis (A); melena (B); systematic hemorrhage manifestations (C); gingival bleeding (D); petechiae (E); ecchymoses (F); and subcutaneous hemorrhage (G). Figure S3: Sensitivity analysis (leave-one-out) for neurological symptoms: systemic neurological manifestations (A); disturbance of consciousness (B); mental state change (C); seizures (D); tremors (E); lethargy (F); coma (G); convulsions (H); and headache (I). Figure S4: Sensitivity analysis (leave-one-out) for complications: disseminated intravascular coagulation (DIC) (A); multiple organ dysfunction syndrome (MODS) (B); acute respiratory distress syndrome (ARDS) (C); heart failure (D); arrhythmia (E); and renal injury (F); S1: PRISMA2020 Checklist.

## Abbreviations

The following abbreviations are used in this manuscript:

SFTS	Severe fever with thrombocytopenia syndrome
SFTSV	SFTS virus
WHO	World Health Organization
ARDS	Acute respiratory distress syndrome
OR	Odds ratio
CI	Confidence interval
RT-PCR	Reverse-transcriptase polymerase chain reaction
NOS	Newcastle–Ottawa quality assessment scale
DIC	Disseminated intravascular coagulation
MODS	Multiple organ dysfunction syndrome
SIRS	Systemic inflammatory response
CSF	Cerebrospinal fluid
DAMPS	Damage-associated molecular patterns
