# Supplementary material for: Clinical Predictors of Mortality in Severe Fever with Thrombocytopenia Syndrome: An Updated Systematic Review and Meta-Analysis
Source: Pathogens. 2026 Jul 21;15(7):767. doi: 10.3390/pathogens15070767 (PMC13415309; doi:10.3390/pathogens15070767)
Supplement: Supplementary file 1 [file pathogens-15-00767-s001.zip › pathogens-4421931-supplementary.pdf]

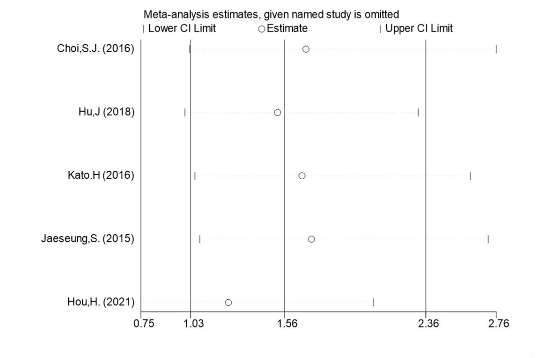

(A)

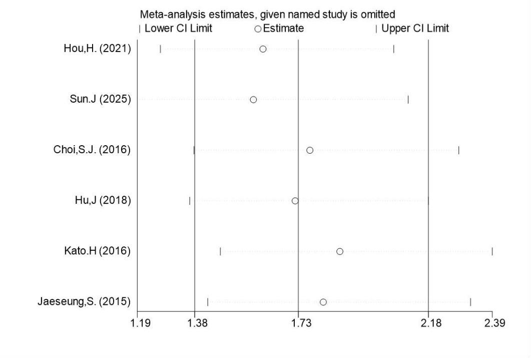

(B)

Figure S1. Sensitivity analysis (Leave-one-out) for underlying diseases: diabetes(A); hypertension(B).

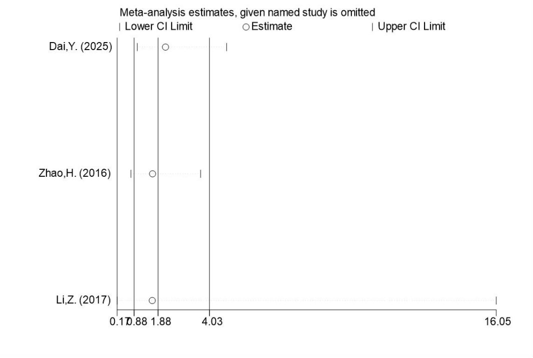

(A)

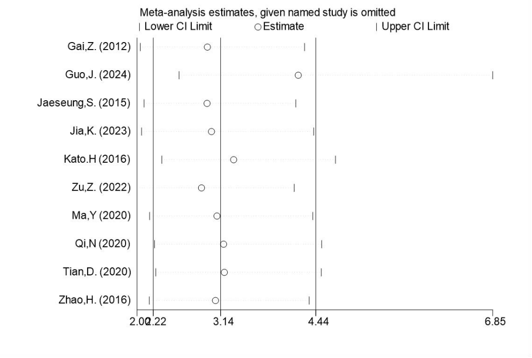

(B)

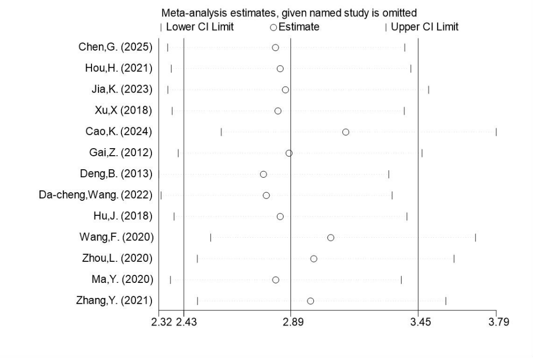

(C)

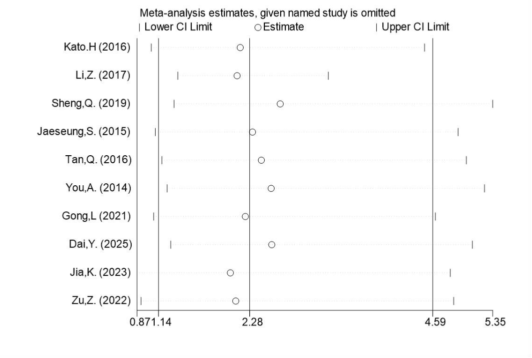

(D)

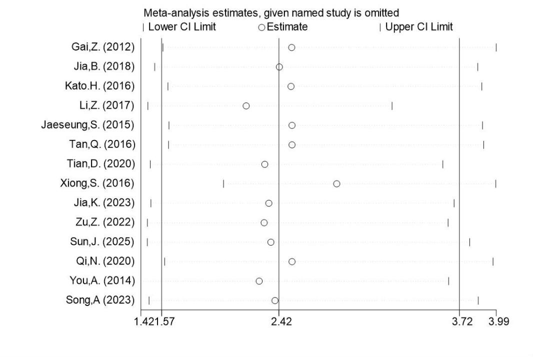

(E)

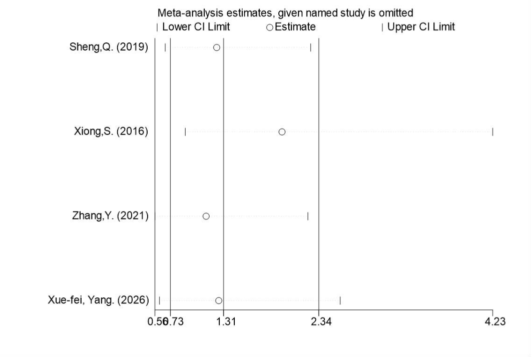

(F)

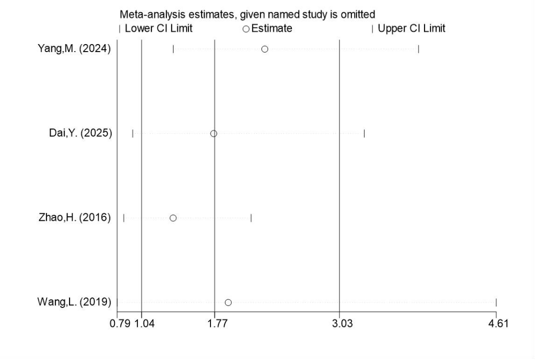

(G)

Figure S2. Sensitivity analysis (Leave-one-out) for hemorrhagic symptoms: hematemesis(A); melena(B); systematic hemorrhage manifestations(C); gingival bleeding(D); petechiae(E); ecchymoses(F); subcutaneous hemorrhage(G).

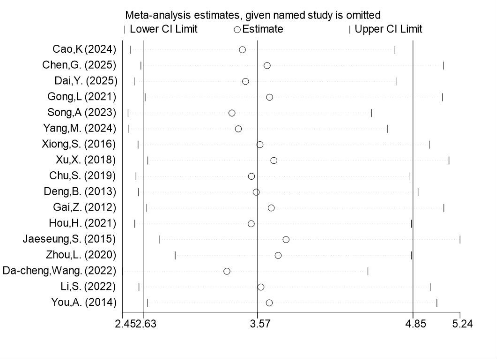

(A)

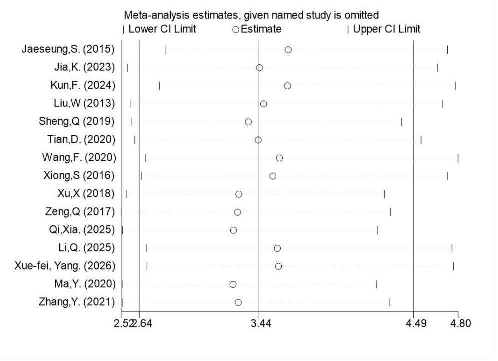

(B)

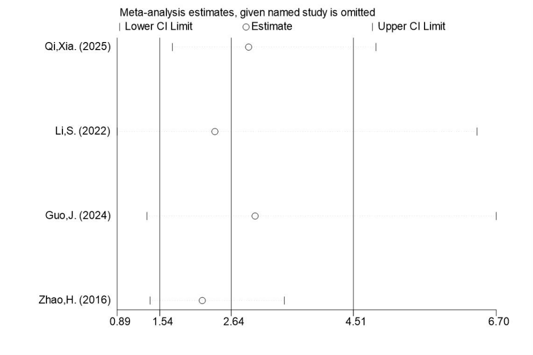

(C)

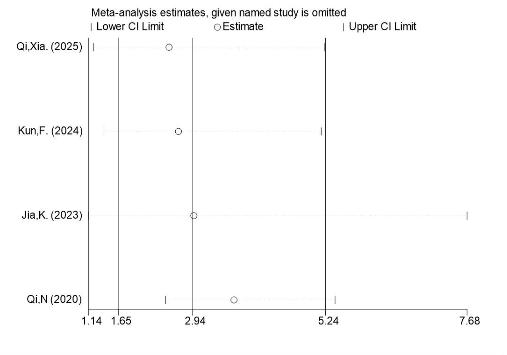

(D)

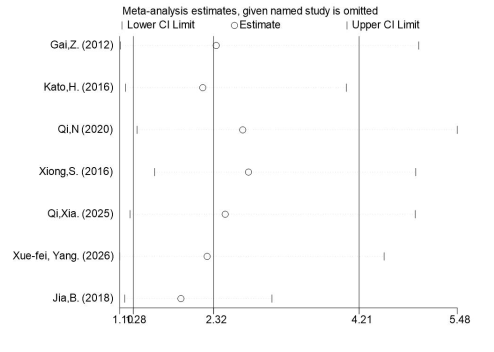

(E)

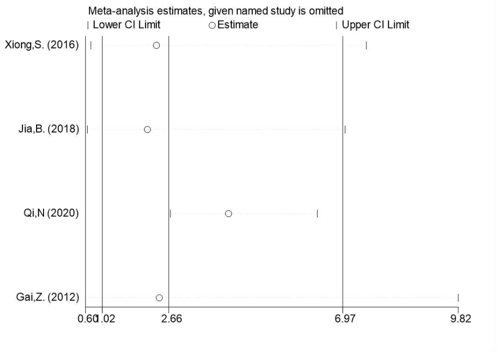

(F)

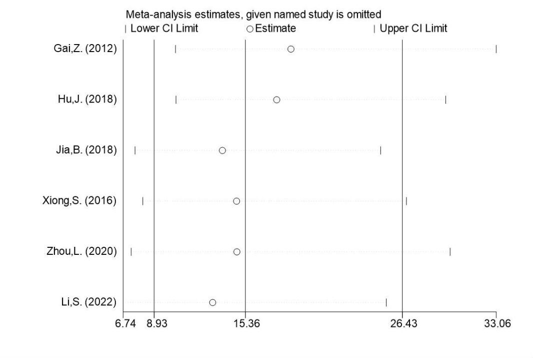

(G)

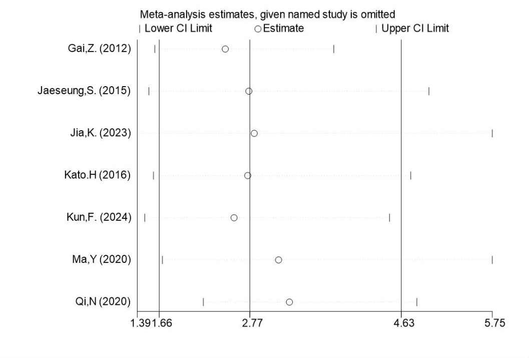

(H)

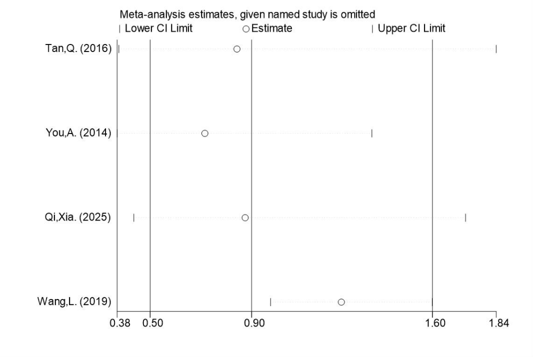

(I)

Figure S3. Sensitivity analysis (Leave-one-out) for neurological symptoms: systemic neurological manifestations(A); disturbance of consciousness(B); mental state change (C); seizures(D); tremor(E); lethargy(F); coma(G); convulsions(H); headache(I).

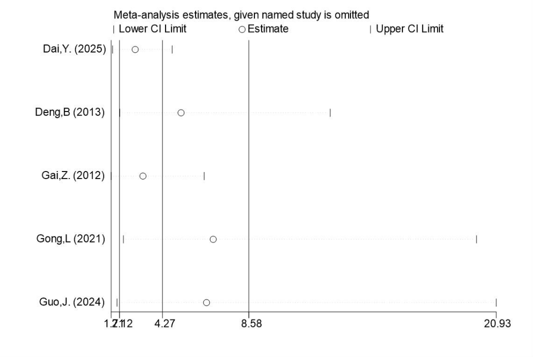

(A)

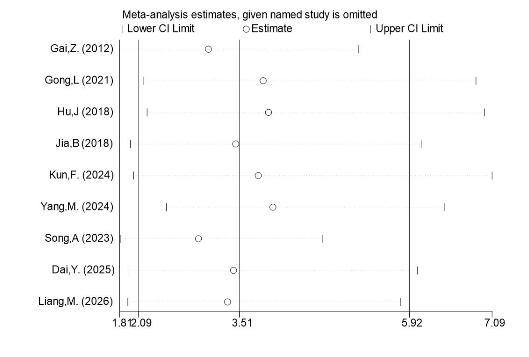

(B)

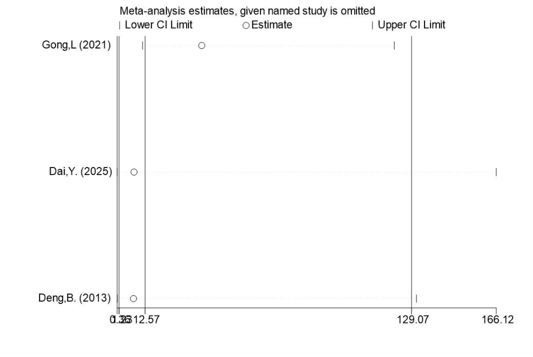

(C)

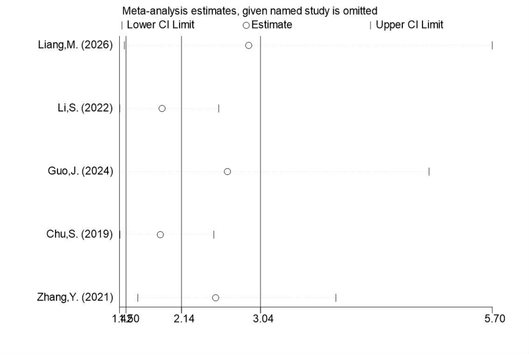

(D)

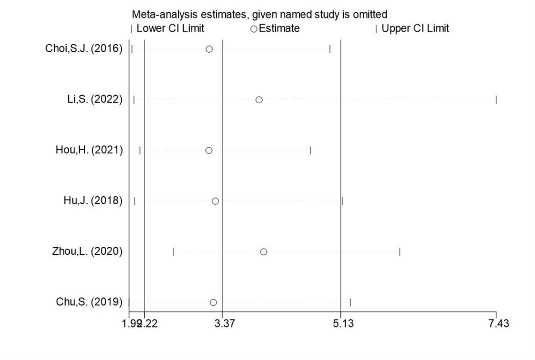

(E)

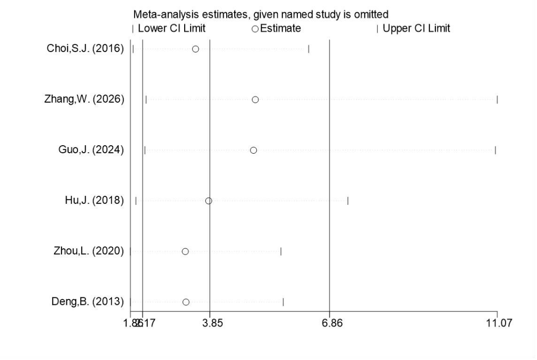

(F)

Figure S4. Sensitivity analysis (Leave-one-out) for complications: disseminated intravascular coagulation(DIC)(A); multiple organ dysfunction syndrome(MODS)(B); acute respiratory distress syndrome(ARDS)(C); heart failure(D); arrhythmia(E); renal injury(F).
